# Rule-Breaking and Rulemaking: Governance of the Antibiotic Value Chain in Rural and Peri-Urban India

**DOI:** 10.3390/antibiotics14121269

**Published:** 2025-12-15

**Authors:** Anne-Sophie Jung, Indranil Samanta, Sanghita Bhattacharyya, Gerald Bloom, Pablo Alarcon, Meenakshi Gautham

**Affiliations:** 1 School of Politics and International Studies, University of Leeds, Leeds LS2 9JT, UK; 2 Department of Veterinary Microbiology, Faculty of Veterinary and Animal Sciences, West Bengal University of Animal and Fishery Sciences, Kolkata 700037, India; drisamanta@wbuafscl.ac.in; 3 Public Health Foundation of India, Haryana 122002, India; sanghita2214@gmail.com; 4 Institute of Development Studies, Brighton BN1 9RE, UK; g.bloom@ids.ac.uk; 5 Department of Pathobiology and Population Science, Royal Veterinary College, London NW1 0TU, UK; palarcon@rvc.ac.uk; 6 Department of Global Health and Development, London School of Hygiene and Tropical Medicine, London WC1E 7HT, UK; meenakshi.gautham@lshtm.ac.uk

**Keywords:** antimicrobial resistance, informal providers, value chain governance, antimicrobial stewardship, India

## Abstract

**Background/Objectives**: Antimicrobial resistance (AMR) is a growing global health challenge, driven in part by how antibiotics are accessed, distributed, and used within complex value chains. In peri-urban India, these supply chains involve a range of formal and informal actors and practices, making them a critical yet underexamined focus for antimicrobial stewardship efforts. While much research has focused on the manufacturing and regulatory end, less is known about how antibiotics reach consumers in rural and peri-urban settings. This study aimed to map the human antibiotic value chain in West Bengal, India, and to analyse how formal and informal governance structures influence antibiotic use and stewardship. **Methods**: This qualitative study was conducted in two Gram Panchayats in South 24 Parganas district, West Bengal, India. Semi-structured interviews were carried out with 31 key informants, including informal providers, medical representatives, wholesalers, pharmacists, and regulators. Interviews explored the structure of the antibiotic value chain, actor relationships, and regulatory mechanisms. Data were analysed thematically using a value chain governance framework and NVivo 12 for coding. **Results**: The antibiotic value chain in rural West Bengal is highly fragmented and governed by overlapping formal and informal rules. Multiple actors—many holding dual or unofficial roles—operate across four to five tiers of distribution. Informal providers play a central role in both prescription and dispensing, often without legal licences but with strong community trust. Informal norms, credit systems, and market incentives shape prescribing behaviour, while formal regulatory enforcement is inconsistent or absent. **Conclusions**: Efforts to promote antibiotic stewardship must move beyond binary formal–informal distinctions and target governance structures across the entire value chain. Greater attention should be paid to actors higher up the chain, including wholesalers and pharmaceutical marketing networks, to improve stewardship and access simultaneously. This study highlights how fragmented governance structures, overlapping actor roles, and uneven regulation within antibiotic value chains create critical gaps that must be addressed to design effective antimicrobial stewardship strategies.

## 1. Introduction

India has one of the highest burdens of bacterial infections in the world: around 25% of child deaths are due to pneumonia, and crude mortality attributable to infectious diseases is 417 per 100,000 persons [1]. In addition, India also has a significant burden of antimicrobial resistance (AMR): in 2019, 297,000 deaths were directly attributed to AMR in India, with another 1,042,500 deaths associated with AMR [2]. India also has the highest burden of antibiotic-resistant tuberculosis in the world [2]. This trend is exacerbated by the high antibiotic consumption in India in response to the high disease burden and a lack of a strong public healthcare system, especially at the primary level [3]. In fact, in 2015, India was the world’s biggest consumer of antibiotics with an estimated 6.3 billion defined daily doses (DDDs) ahead of China (3.8 DDDs) and the United States (2.9 DDDs) [4]. While per capita consumption remains considerably lower in low- and middle-income countries (LMICs), the DDD in India per 1000 population in 2018 was 14.2, which signifies a drastic increase from 2000 only 6.3 DDD/1000 population [5].

Implementing effective regulations to contain AMR in LMICs, like India, is particularly challenging due to the pluralistic nature of the health system and the inadequacy of regulatory frameworks and their enforcement mechanisms. India’s health system is characterised by a public sector that, despite its limitations of human and financial resources, provides essential care to a significant portion of the rural population, which makes up 68.8% of the country’s population, and a far larger private sector, where over 70% of people seek primary healthcare [5,6,7]. As such, antibiotic supply chains can be divided between those where distribution is fully operated via government services, with end points being the hospitals or the Prime Minister’s Indian public pharmacy initiative (Pradhan Mantri Bhartiya Jan Aushadhi Pariyojana), and those operated by private stakeholders who distribute antibiotics to pharmacies and informal providers (IPs). Many private providers, however, lack formal medical qualifications, and rural and small-town suppliers represent a substantial portion of the pharmaceutical market, with antibiotics generating large profits [8]. Similar findings have been documented in work studying the provision of livestock healthcare [9].

The primary aim of any public health regulatory system is to protect populations from health risks, such as dangerous or ineffective medicines and harmful medical practices, but this goal can clash with the need to provide widespread access to health goods and services in an under-resourced system [1,10]. While the sale of prescription drugs without proper authorisation is banned, it remains a common practice, particularly in rural areas, where the regulatory framework is weak, and enforcement is difficult [11]. Antibiotic stewardship is further hindered by knowledge asymmetries and complex value chains [9,12,13]. Despite the recognition of these dangers, the health system’s dependence on IPs and retail shops makes regulation difficult to enforce. Consequently, while AMR is a growing concern, people’s lack of proper (formal) access to essential medications is an equally significant concern.

Although there is a growing body of global scholarship on antibiotic value chains and AMR governance, most existing studies emphasise manufacturing, distribution, or livestock systems, with far less attention to how community-level supply chains operate in LMICs and how they intersect actors situated at the ‘top end’ of such chains. Within this limited body of work, studies suggest that multiple actors in the antibiotic value chain contribute to misuse. These actors include medical representatives who incentivise antibiotic use [14], wholesalers, distributors, and retailers who sell antibiotics and encourage inappropriate consumption [15], and pharmaceutical companies with vested commercial interests in rural health markets. Building on this literature, our study addresses an evidence gap about the functioning and governance mechanisms of value chains that supply antibiotics to providers and consumers at the retail end in emerging rural and peri-urban markets in India. This knowledge is needed to strengthen the governance of the chains around principles of antibiotic stewardship. In doing so, our analysis not only documents the functioning of these networks but also examines where and how stewardship interventions might most effectively be anchored within them.

This study therefore contributes new empirical and conceptual insights by tracing the full antibiotic value chain in rural and peri-urban West Bengal and identifying the multiple governance roles played by wholesalers, distributors, and medical representatives. By examining these actors as part of a broader governance system, we highlight how antimicrobial stewardship (AMS) could be designed differently, focusing not only on informal providers but also on the upstream market actors who shape their practices.

To start addressing the misuse of antibiotics and promote AMS, this study aimed to map antibiotic value chains and markets in rural and peri-urban areas in West Bengal, India, and examine their composition, interactions, and formal and informal regulations shaping these chains. The study focuses only on the value chain operated by private stakeholders, which is the one use by informal providers; we do not analyse the governance of the value chains operated by the government. By analysing how antibiotics move through formal and informal systems in West Bengal, this paper highlights the fluid and overlapping roles of actors across the value chain and argues that effective antimicrobial stewardship requires shifting focus beyond IPs towards higher-level actors such as medical representatives, for example, whose practices and influence are central to shaping antibiotic distribution and use.

## 2. Governance Framework for Value Chains

A value chain is described as the production, procurement, and distribution of a product, starting from the manufacturer and ending with the consumer, and includes operations, finance, and marketing [16]. An analysis of value chain activities goes beyond a description of the different functions within it and examines the dynamics of the interlinkages and relationships within the chain as well as the value added along the nodes of the chain. This makes a value chain analysis a valuable tool for our study, since it makes it possible to analyse the relationships between informal and formal workers, rather than seeing them as separate and disconnected actors.

A key feature of a value chain analysis is the examination of governance. This involves understanding the actors who form links in a value chain, their relationships and interactions, and who influences whom, i.e., their relative power. A value chain analysis embraces the co-ordination processes, mechanisms of power, and influence in the chain, agreements, and rules that exist between the different links in the chain. Matters concerning who decides what commodities are produced, why particular stakeholders interact, what type of rules exist (whether these are legislation, private standards, or informal organisational and cultural norms), how these are enforced and codified (including incentives, agreements, and sanctions), and who are the rule makers, implementors, and enforcers in the system, can all be studied through the analysis of who governs value chains and markets [17,18].

By adapting and complementing Kaplinsky and Morris’ [1] value chain governance framework, this paper seeks to analyse value chains according to five dimensions (Table 1). First, it seeks to identify relevant actors throughout the value chain and their relationships to other members of the chain. As Kaplansky and Morris [1] specify, “coordination usually … requires monitoring of the outcomes, linking the discrete activities between different actors, establishing and managing the relationships between the various actors comprising the links, and organising the logistics to maintain networks of a national, regional or global nature”. The second element describes the nature of actor relationships, why actors seek out specific relationships and how this influences the value chain. This includes “identifying dynamic rent opportunities and apportioning roles to key players which reflects an important part of the act of governance” according to Kaplansky and Morris [1]. The third element consists of understanding the formal regulations that define the remits of the value chain, or as Kaplansky and Morris called it, legislative governance [1]. Fourth, we analyse how these formal regulations are supplemented, omitted, or weakened by informal mechanisms of governing interactions. This is where we add to Kaplansky and Morris’s understanding of governance, which focuses on firms and governments as primary units of analysis. However, we argue that this does not reflect the realities of the antibiotic value chain, where much of the distribution in India depends on informal providers. Finally, we analyse how the different actors make, break, and negotiate rules and how rules are enforced (power and legitimacy of actors). Combined, these elements allow us to understand challenges and opportunities for antibiotic value chain governance and antimicrobial stewardship in West Bengal’s antibiotic and healthcare market.

## 3. Methods

### 3.1. Study Setting

This study was part of a larger research project on the drivers of antibiotic use in community settings and the design of fit-for-purpose antibiotic stewardship interventions. The study was located in West Bengal in eastern India, a medium development state. West Bengal, with its population of 91 million, is India’s fourth most populous state and has a per capita net state domestic product slightly below the national average [19]. A recent survey highlighted a significant burden of infectious diseases, with 16% of the population reporting acute illnesses such as fever, respiratory infections, diarrhoea, and malaria [20]. Previous studies, including our own, have documented inappropriate antibiotic dispensing by local healthcare providers, many of whom are informally trained [5,9,21]. Given that community expectations and perceptions heavily influence provider practices in West Bengal, it was deemed appropriate to conduct a focused community-based study in this region to better understand and address these issues.

The two specific study sites were selected from South 24 Parganas, a medium-sized development district in West Bengal with a population of seven million, 84% of whom live in rural areas. Two Gram Panchayats (GP), which are village administrative units, GP-A and GP-B, were chosen in a purposive manner based on number of households keeping livestock, number of medical IPs in the area, and the GPs’ proximity to Kolkata, the capital of the West Bengal state. GP-A was located 60 km south-west from Kolkata (approximately two to three hours by road); GP-B was located further from Kolkata (95 km south-east, approximately four to five hours by road and ferry). Variation in the sites’ proximity to Kolkata was intended to enhance the generalisability of the findings by capturing differences in antibiotic access, distribution, and governance across peri-urban and more rural contexts. The number of informal medical providers at the study sites was considered based on the hypothesis that overlaps might exist between human and livestock antibiotic provision—an occurrence reported in several LMICs [22]—but, at the time of this study, not yet documented in India. Both value chains were investigated under the broader project to design effective One Health antibiotic stewardship interventions in community settings.

### 3.2. Data Collection, Management and Analysis

Details of the data collection are described in the parallel study of the animal antibiotic value chain [9]. To summarise, a team of four researchers, trained in qualitative field data collection, interviewed key informants in the two study sites between June 2019 and January 2020. We interviewed a total of 31 key informants from GP-A (n = 13), GP-B (n = 15), and the district and state level (n = 3) including informal and formal primary healthcare providers, medical representatives, pharmaceutical managers, stockists, wholesalers, pharmacists, leaders of IP associations, a senior academic, and drug inspectors. Interviewees were selected using purposive convenience sampling using a network of partners in the project and a snowballing technique with the aim of capturing perspectives from all actors operating in the value chain. Many participants were selected based on their expert knowledge of the system, while others were selected to obtain individual stakeholder perspective. For example, drug inspectors have an in-depth understanding of the type of drug sellers in the GPs and their connections within the chain. They are ideally situated to explain the governance of the chain. Similarly, IPs association officials are very knowledgeable of the IPs’ practices. Managers of large pharmaceutical companies are well-aware of the governance of the production sector. Where possible, such experts at each node of the chain were selected. When this was not possible (e.g., for wholesalers), several individuals were interviewed to understand specific parts of the chain. Interviews were conducted until information saturation was reached.

Information gathered from a review of existing literature was used to generate a priori interview guides for the semi-structured in-depth interviews. These guides were designed to differentiate the types of stakeholders involved in the antibiotic value chain, their roles, inter-actor relationships, and use of antibiotics. Key informants were asked to describe their role in the value chain and provide an overview of how they interact with each other, in relation to antibiotics. This provided an opportunity to gain an understanding about each participant’s awareness of antibiotics as treatments, as this variable was expected to vary across informants. During each interview, key informants were asked to engage in a participatory exercise to produce antibiotic value chain maps, in which interviewees identified all nodes and actors known to them. Interviewees were asked to categorise each actor identified according to their method of operation.

This differentiation of stakeholders was then used to prompt questions about differences in terms of suppliers and clients or users of antibiotics and also gather their perception of how these nodes and actors are connected. In addition, participants were asked to indicate where the major flows of antibiotics occurred, what regulations they were aware of and what regulations were followed across the different nodes in the chain. Where possible interviewees were asked to provide information about which antibiotics they most commonly dealt with, and their perceptions of antibiotic resistance.

Most of the interviews were conducted in English. We targeted people with high-level knowledge of the chain, and these people have good command of the English language. In addition, members of our research team who are native speakers of the interviewee’s first language were present for situations where clarification was needed. For stakeholders with no or limited knowledge of the English language, interviews were conducted in the interviewee’s preferred language (Hindi or Bengali) and translated verbatim by a research team member who is a native speak of the language and knew the interview guide and project objectives.

Interviews were conducted for 45 to 90 min, and participants were able to choose the location of the interview, which usually took place at their workplace. All interviews were conducted face-to-face following interviewees’ signed consent. Interviews were conducted in Bengali, Hindi, or English. All interviews were transcribed and translated into English verbatim. We acknowledge the potential for translation bias and sought to mitigate this by working closely with bilingual local partners who reviewed transcripts and notes and clarified key terms. We used a thematic data analysis approach according to the governance framework, which applies both inductive and deductive reasoning. The first and last authors collectively coded a sample of transcripts before the first author conducted a line-by-line coding and analysis of all interview transcripts, using NVivo 12 qualitative data analysis software (QSR International Pty Ltd., Burlington, MA, USA, Version 12, 2018). Researcher positionality and potential bias were carefully considered throughout the process, and reflexivity is discussed further in Section 6.

To ensure analytic rigour, we combined deductive and inductive coding strategies. The five governance dimensions adapted from Kaplinsky and Morris [1] formed the deductive framework and guided our initial coding structure. During the first round of coding, two researchers independently coded a subset of transcripts to refine this initial framework and identify inductive codes that captured unanticipated but salient themes emerging from the data. Examples of such inductive themes included trust, learning, training, omission of regulation, and licence sharing. These codes were discussed in several rounds of team meetings and consolidated into a shared codebook. The codebook was iteratively refined as additional transcripts were coded and new nuances emerged. Inter-coder reliability was addressed through discussion of discrepancies until consensus was reached, rather than by formal calculation of agreement metrics, which is consistent with qualitative research practice.

We used a thematic analysis approach structured around the governance framework, combining inductive and deductive reasoning to interpret the data. Themes were derived by comparing coded segments across stakeholder groups to identify recurrent patterns, contradictions, and relationships between governance practices and antibiotic flows. Emerging interpretations were revisited against the original transcripts to ensure grounding in participants’ accounts.

The study design was reviewed by and received ethical approval from institutional ethics committees of the London School of Hygiene and Tropical Medicine (UK) and the Public Health Foundation India (India).

## 4. Results

**Figure 1:**
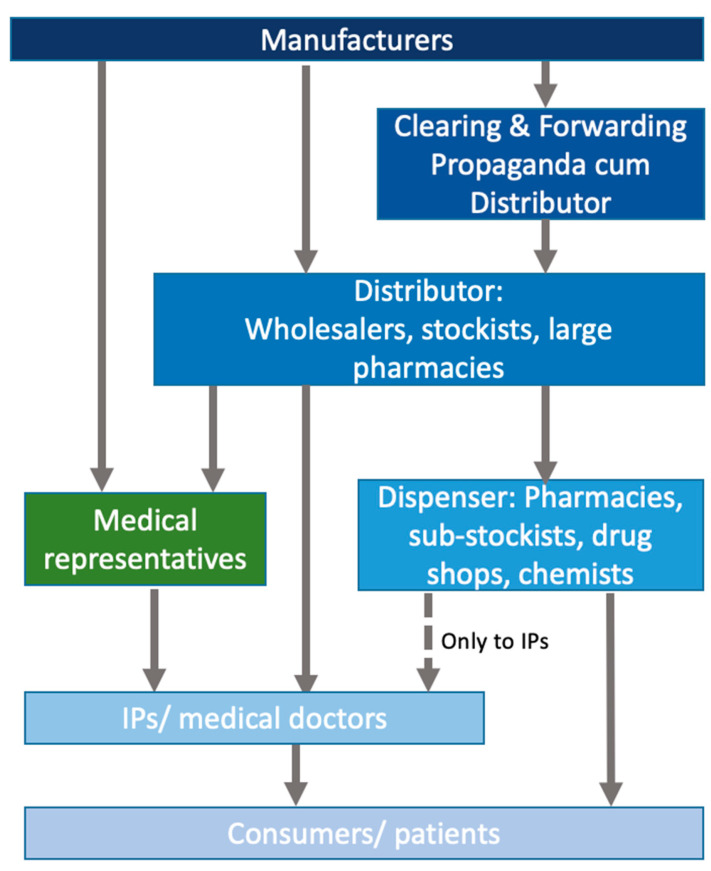
Stylised overview of the antibiotic value chain in the private sector in peri-urban West Bengal.

Based on interviews with key informants, we constructed descriptions of the key actors and their interactions and relationships in the antibiotic value chain in rural and peri-urban areas of West Bengal, starting from the point of manufacturing and proceeding to the end consumer. Figure 1 provides a simplified overview of the chain, which will be described in more detail in this section.

### 4.1. Actors and Actor Relationships

The interviews revealed that the antibiotic market in our study sites was densely populated by a complex network of diverse private sector actors and a multi-layered drug distribution chain, spanning four to five tiers. At the top were the pharmaceutical manufacturers with their specific clearing and forwarding agents. The second rung was made of stockists and wholesalers, some of whom had sub-stockists attached to them who were based closer to villages and supplied communities and informal providers. The final rung of the value chain consisted of a diverse group of small retailers including chemists/pharmacies and drug shops or/and informal providers. The latter dispensed, at a price, antibiotics to end consumers—patients and those who sought treatment for animals. While these distinctions suggest a neat taxonomy, they were more ambiguous in practice since the different actors could fall within multiple groups as they took on more than one role within the value chain. We describe these actors and their roles below. The value chain for antibiotics used in livestock has been visually depicted in our study focusing on livestock and overlapped with the human supply chains [9].

#### 4.1.1. Pharmaceutical Manufacturers and Their Distributing Companies

Interviews with pharmaceutical managers and medical representatives revealed that there were numerous domestic pharmaceutical manufacturers; nearly 88% of the Indian pharmaceutical market consists of micro, small, and medium enterprises [23]. One medical representative estimated that there were around 70 small drug manufacturers in West Bengal licensed by the state government. Many small drug manufacturing companies sold drugs to licensed drug distributors, while others sold their drugs to primary healthcare providers (formal and informal), directly employing medical representatives to reach them. As one medical representative observed, “every day there is a new company being launched. And they will have antibiotics because antibiotics have a big market” (GP-B_MedRep_2).

While some companies produced their own active antibiotic ingredients, others imported these depending on the scale of their operations. Large manufacturers that produced active pharmaceutical ingredients were relatively few; most small-scale producers only produced formulations. However, the boundary between the two kinds of company was not clear-cut. Small manufacturers often acted as auxiliary producers for larger ones, formulating drugs and packaging them under large company names through ‘loan licences’ or subcontracting agreements. These agreements allowed a smaller company to produce a product owned by another firm.

Loan licensing and subcontracting were commonly used to reduce costs, incl. excise duty, sales tax, or labour since small-scale manufacturers operated under more favourable regulatory and wage conditions. The quality of the drugs, however, was reported to be comparable to those produced by large manufacturers, supported by strong connections between well-known global pharmaceutical companies and local ones. As a professor of pharmacology explained, large multinational firms often developed products in-house but outsourced production to smaller local manufacturers through loan licensing or third-party arrangements. The multinational retained responsibility for marketing, while the local company held the manufacturing licence, creating an efficient division of labour that combined global branding with local production capacity.

Yet, most pharmaceutical companies based in India were small- and medium-sized enterprises which usually did not have their own marketing department or distribution system. Consequently, they relied on Propaganda cum Distributor (PD) companies and Clearing and Forwarding (C&F) agents for marketing and distribution. PD companies were pharmaceutical franchise companies which managed the products, new launches, decided brand names, promotional material, and point of sale material. PD companies were attached to a drug manufacturer. In contrast, C&F agents were independent companies, or businesses, and a type of super-stockist that facilitated and oversaw a drug manufacturer’s distribution of medication. For India, the number of C&F agents and PD companies was difficult to obtain; the definition and roles of both types of companies reflected tax and licensing conditions, which change frequently. One medical representative described C&F agents as regional intermediaries that received stock from manufacturers, handled onward distribution, and earned a commission, typically between 2% and 5%, based on the volume of sales, without the need for direct maintenance of retail operations.

#### 4.1.2. Stockists and Wholesalers

Stockists and wholesalers made up the large and diverse middle rungs of the value chain and connected the C&F companies with retailers and prescribers/dispensers. The distinction between ‘stockist’ and ‘wholesaler’ was not always clear-cut, and the terms were often used interchangeably. This ambiguity was further compounded by the fact that both operated under the same licence—a wholesaler’s licence. This licence legally permitted them to sell drugs only to retail businesses.

The key difference was that wholesalers were often larger, with wider outreach, who stocked drugs manufactured by several different pharmaceutical companies. As explained by a medical representative, stockists typically dealt with a limited number of C&F agents and supplied goods directly to retailers, whereas wholesalers handled products from many different manufacturers, maintaining larger inventories and wider distribution networks. Retailers preferred wholesalers because they could obtain a complete range of stock from a single source.

Stockists, on the other hand, were often tied to one pharmaceutical company or C&F agent. As one key informant explained, C& F agents could have 50–200 stockists in every state who are then sent out to the pharmacists or chemists. Both wholesalers and stockists sold drugs to a diverse group of actors, including retailers as well as physicians and IPs, and in a few exceptional cases to patients directly. Medication sold to patients directly included antivirals, tuberculosis medication, and cancer medication.

There were other finer differences as well, as we learned through the interviews. Wholesalers were usually situated closer to cities, whereas stockists, and especially sub-stockists, were located closer to their customers. Wholesalers were divided into two types: one type was situated in cities and peri-urban areas with branches at more rural levels. These wholesalers sold drugs to pharmacy retailers located in rural and urban areas, charitable dispensaries, and to IPs through their own medical representatives. The other category of wholesalers was only situated at the block level and procured antibiotics (generic and branded) from the Kolkata wholesale market. This group sold only to IPs and small retailers.

Stockists, in contrast, did not have their own medical representatives, but had sub-stockists. These sub-stockists were individuals with local ‘shops’ (small retail shops) where IPs could purchase their medication. Sub-stockists could also be medical representatives or even IPs who would stock certain drugs and supply them to a group of their own type of local providers. Given the large number of wholesalers and stockists, competition for customers was fierce within these groups. Stockists and wholesalers outbid competitors through price dumping, binding regular customers through better credit conditions, or price reductions (see credit system). Wholesalers’ medical representatives were often rewarded by receiving a share of the profits they make, and medical representatives were known to bring presents when they visited IPs and formal providers.

An emerging novelty was online pharmacies (only appearing over the past 5 years). These digital pharmacies worked through a mobile app where patients uploaded prescriptions, and the medication was delivered from the wholesaler directly to the patient without any further retailers in between. Buying drugs through online pharmacies was said to be 10–15% cheaper than through a physical retail shop.

#### 4.1.3. Medical Representatives

Besides selling drugs to licensed drug distributors, most manufacturing companies and several stockists employed medical representatives to target informal providers, formal primary care physicians, and retailers. In contrast to stockists, large pharmaceutical companies usually trained their medical representatives specifically for the drug they were tasked with selling, rather than providing them with training on the active ingredient more generally or with broader pharmaceutical knowledge. Medical representatives were often described as ‘middlemen’ between companies whose products they sold, primary care physicians/providers, and chemists. Their role was to advertise and promote their company’s products. Medical representatives promoted a specific drug to a physician, but they subsequently also informed nearby “chemists (retail pharmacist) that the physician in their close proximity would now prescribe this specific medication and that the chemist ought to stock it” (GP-B_MedRep_1).

In practice, medical representatives act as mobile connectors—monitoring the needs of chemists, relaying those requests to stockists, and ensuring that the right products are delivered—thus creating demand rather than simply moving inventory along the chain. Their close ties to informal providers were reinforced by leaving free samples as a promotional tactic, according to our interviewees.

#### 4.1.4. Drug Retailers, IPs, Primary Care Physicians

Terms such as retailer, pharmacist, and chemist were often used interchangeably to refer to a retail drug shop that was licensed to sell drugs, including antibiotics. Besides IPs, pharmacists/chemists/retailers were also an important first point of contact for healthcare in peri-urban and rural areas (though less so in rural areas, where IPs doubled up as pharmacists). Pharmacists provided diagnoses and medicines (often including powerful prescription drugs) without the intervention of any other kind of practitioner. Most small hospitals and nursing homes also had in-house pharmacies, and many required patients to buy the drugs on the premises, whether they were in- or out-patients. Retailers, chemists, and pharmacies varied widely in size and were operated by individuals with diverse levels of medical knowledge. For a retailer to be licensed, the shop needed to have one licensed pharmacist present at all times. Tertiary care hospital chains, like Apollo, and large corporations were said to have better-regulated pharmacy chains that required prescriptions. However, these were situated in cities and not in rural areas.

In the rural interiors, it was IPs who provided case management and dispensed antibiotics, thus doubling as chemists. They filled a crucial gap in the formalised healthcare system here, as in other parts of India. They were trusted members of the community and seen as a friend or a friendly next-door neighbour who was also knowledgeable of the socio-economic background of their patients. They were also seen as affordable and effective as they provided powerful drugs, including antibiotics.

If they did not have certain drugs in stock, they would send their patients to the nearest retailer. They were also connected to physicians in cities which meant that they could refer severe cases to physicians and negotiate priority appointments for their patients in overcrowded hospitals. In remote settings, IPs were seen as a relatively inexpensive and often the only way of accessing essential medication without long, expensive travel, sometimes even for maternal emergencies. As one medical representative explained, without the presence of RMPs (a term commonly used for IPs), healthcare in remote villages would collapse. In many interior parts of districts like South 24 Parganas, poor transport and communication mean that patients—especially women in labour—cannot reach urban hospitals in time. For these communities, RMPs remain the only accessible and dependable source of basic treatment and emergency care.

Yet, at the same time, an IP’s practice was also their livelihood, often placing an IP in a position where they had to navigate and balance their own business interests with the patient’s wellbeing, as well as the patient’s expectation as a paying customer. A participant IP who was also a sub-stockist explained his business model vis-à-vis his relationships to patients. He explained that using high-priced branded medicines such as those from Ranbaxy or Alembic limited his ability to offer discounts to patients, as these products had to be bought with cash and offered little room for credit. Yet, because he lived within the same community, his patients—many of whom had limited means—expected some form of concession or flexibility when purchasing medicines. This dynamic, he suggested, often created tension between maintaining his business viability and meeting community expectations.

The interviewed IP shines a light on the complicated relationships within the value chain. The IP procured branded medication for which he has to pay in cash, which he may not have. For the IP to be able to do so, their patients have to pay in cash too. However, this sometimes went against patient expectations and their financial abilities.

IPs did not have a formal medical qualification, but basic schooling and some post-school diplomas and certification. They learned by assisting primary care physicians or working in hospital facilities before opening their own practice. IPs had strong associations that protected their interests and negotiated with the government on behalf of members. The government of West Bengal was attempting to recognise them as community health workers following a six-month training programme, which still did not allow them to dispense antibiotics or other prescription drugs, however. Nonetheless, IPs dispensed a wide variety of drugs and antibiotics and received continuing medical education and training by pharmaceutical companies and medical doctors and hospitals.

### 4.2. Nature of Interactions

#### 4.2.1. Market Dynamics

A large part of interactions between value chain actors were based on market dynamics and involved business relationships. Prices, credit, and discount systems along with logistical concerns (proximity, delivery, ability to reach a specific location) influenced the choices for where drugs including antibiotics were procured. Drugs were generally purchased in cash or on credit and all actors along the value chain had credit and discount systems in place, besides the manufacturing and pharmaceutical companies. Credit and discount systems were not formally regulated and there were no clear or universal ‘rules’ regarding how much credit or discount was given at different rungs.

Credits and discounts were agreed to by purchaser and seller with varying degrees of formality/informality in terms of repayments, credit duration, or amount of discount. In interviews, IPs highlighted the importance of pricing and the availability of credit and discount schemes when choosing which wholesaler or stockist to buy medication from. Similarly, one of the main drivers for patients to seek help from informal providers was that they only paid a charge for the medicines, which they could pay in cash or on credit. How regularly a supplier or provider bought drugs, and in which quantities, depended on the overall size of the business. For example, IPs only purchased small amounts irregularly, according to key informants.

While selling drugs and charging consulting fees provided a livelihood for IPs, patients also expected certain benefits from visiting IPs vis-à-vis a professionally trained physician. One of these was that patients expected prices to be lower when purchasing drugs from their local IP than when visiting a physician. A participant elaborated on the dilemma of helping patients, market pressures, and patients’ demands:

Because for the rural healthcare providers (RHCP) government rule is ‘you cannot keep antibiotics’. But if I do not keep antibiotics, I have to close the chamber and stay hungry. I have to give some antibiotics. I will not use antibiotics for all cases, but that does not mean I cannot use antibiotics in any case. If I do not use antibiotics for any case, why should [patients/villagers] come to me? … I provide full course [of medicine] and use that much [of medicine/antibiotic] which is needed to sustain my livelihood. (GP-B_Stockist_2)

The monetary aspect and concern for distance to the distributor were determining factors for all actors within the chain. One pharmacist explained that they choose certain products over others because one wholesaler sent a medical representative to market his products, while the others did not. This made it more convenient for the pharmacist to buy products. The product itself, whether it was a generic or branded medication, did not matter in this case if the discount was the same.

Indeed, the value chains were organised around discounts, profits, and credit payments. Credit and discounts were an integral part of the value chain, beginning with IPs and their patients: IPs were conscious of their patients’ financial restraints and offered medical consultations and drugs on credit where patients could not afford the service otherwise. In return, IPs relied on distributors—whether these were retailers, stockists, or wholesalers—to similarly grant them credit. Some credits needed to be paid after 15 or 30 days, for others, a customer (whether an IP or any other retailer) needed to pay the previous bill before they could open another one. In yet other cases, credits had to be paid by the end of the business year. This was especially true for IPs and their patients.

Similarly to the credit system was the system of discounts. Different shops allowed for different discounts and/or credit, which was mainly based on the amount purchased. The ‘higher up’ in the value chain that IPs bought their drugs (from wholesalers, rather than sub-stockists), the lower the prices would be, as ‘middlemen’ and other salespersons were circumvented in the chain. According to interviewees, discounts for various drugs, including antibiotics, varied between 10 and 30% for informal providers and up to 50% for formal providers (GP-B_IP_2).

The difference in discount level was particularly true for medical representatives who visited both formal and informal providers. One medical representative disclosed that he only gave discounts for branded medication, but not for generic drugs. However, in contrast to other key informants, this medical representative explained that formal providers often expected gifts as incentives whereas IPs were less interested in gifts and preferred discounts, so they had larger profit margins (GP-A_MedRep_1). Our interviews showed that discounts and recruitment through presents or free samples are not a fixed system but evolve dynamically according to who is buying or being visited. Differences in practice may also vary according to the practices within a district or geographic area.

Market dynamics shaped both the ways that prices were calculated for generic drugs and so-called branded drugs. Branded items were drugs distributed under the name of a specific company and under a protected name, increasing their market value. Generic drugs were comparatively cheap, allowing for greater profit margins. Most IPs sold both types of medication; however, one wholesaler noted that the profit margins for generic medicines were several times higher than for branded equivalents, meaning that IPs often inflated the retail price of generics in order to generate income.

A medical representative disclosed that they offered up to 50% discount on the drugs they sold. They described a typical pricing structure in which both the representative and the prescriber retained portions of this discount as profit, while the manufacturing company’s share reflected only a fraction of the final retail price. Such arrangements highlight the complexity of drug pricing and the multiple layers of profit embedded within the distribution system. While many IPs named the wellbeing of members of their community as their priority, it is also true that they relied on the income through selling and dispensing medication, including antibiotics.

#### 4.2.2. Learning and Training

Focusing on the bottom end of the value chain, we explored the learning systems that supported knowledge development for IPs. While IPs did not undergo formal medical training, these providers were nonetheless keen to obtain medical knowledge on (new) drugs and how to use them by drawing on their networks within the value chain. As such, medical representatives, formal providers, and important medical sources through which IPs learned about new medicines. As a result, knowledge exchange was centred on learning of new drugs and how to use them, rather than developing foundational skill sets.

The relationships between IPs and formal providers were characterised by learning, training, and mentoring. Several IPs explained that they had shadowed a formal primary care provider for several years before opening their own practice. Many of them still maintained loose ties to known physicians. These connections allowed them to refer patients to trusted doctors in emergencies or to seek advice when necessary. When referring patients, some IPs would request permission to observe the consultation to enhance their knowledge. When patients returned from consultations with formal providers, IPs would study their prescriptions and replicate them the next time they saw a patient with similar symptoms. As one informal provider described, they often learned about new medicines or antibiotics during hospital visits or by examining the prescriptions issued by doctors, using these as models for their own practice.

While this process offered IPs opportunities to expand their medical knowledge, it also meant that the rationale behind prescriptions or dosages was not always explained or fully understood. A professor of pharmacology explained that IPs often emulate the prescribing patterns of formal medical doctors, replicating their prescription practices without fully understanding the underlying clinical reasoning. In addition, IPs valued medical representatives not only for discounts, credits, and the convenience of having them visit, but for their knowledge on the medications they sold. IPs received information on (new) medication from medical representatives, who, in case of new drugs, often also provided samples of the new medication. As such, the relationship between IPs and medical representatives was driven by market dynamics as well as the perceived learning benefits.

#### 4.2.3. Personal Relationships

Most analyses of personal relationships and trust in the health sphere focus on the dyadic practitioner–patient relationship and generalised trust of patients in healthcare providers and systems. However, personal relationships play a role in the value chain too. If an IP or the IP’s mentor and predecessor had purchased drugs from a specific stockist, retailer, or wholesaler, then, one IP reported, the IP would also be inclined to continue this relationship. Similarly, a stockist might be inclined to procure medication from a specific company or distributor because of personal connections as illustrated in the following, “I have a 10-year long relationship with the proprietor of X Enterprise. He said, “We will come regarding a company”. Eventually I started business (with them)” (GP-B_Stockist_2).

Trust also played a role in the choice of drugs purchased by primary care physicians and retail shops. Some actors along the value chain did not feel confident about the quality of generic drugs. This was based on personal experience by prescribers (formal and informal) arguing that some generic drugs proved to be less effective than their branded counterparts. One pharmacist emphasised that “there was a huge difference in the quality” (GP-B_Pharmacy_1), which is why he only stocked branded medication.

### 4.3. Formal Regulation

The regulatory landscape governing antibiotic supply in India is shaped by multiple formal rules intended to control access and ensure quality. However, these regulations are frequently circumvented through informal practices at every level of the value chain, as summarised in Table 2 below. There were two major formal regulations that shaped drug and antibiotic value chains in different ways. One is the Goods and Services Tax (GST), introduced in 2017. Under this Act, only actors holding a GST registration number could purchase drugs from companies and wholesalers. For example, since its introduction, only stockists with GST registration were allowed to purchase prescription drugs from Bagri Market, one of West Bengal’s largest medicine markets. Through our interviews, we learned that the GST system had streamlined drug sales, but only to an extent. Businesses worth above 40 lakhs (ca. GBP 34,000 give in US$) had to register for the GST. Companies must keep a separate accounting system for GST-registered businesses to that for customers without such registration. Buyers who were not GST-registered were registered through a Permanent Account Number—a document issued by the Income Tax Department. Buyers who did not hold either of these registration numbers were not allowed to procure prescription medication because of their informal nature. Yet, retailers continued to sell prescription medication to IPs, thereby introducing a third register—that of an ‘unregistered sale’.

However, while the GST had introduced stricter oversight mechanisms, it had also complicated procedures to upload stock information and hampered the return of expired antibiotics. Companies and C&F agents were no longer allowed to accept expired medicines to block any illicit attempts at redistributing expired drugs. In so doing, supply chain actors no longer had a means of disposing of expired medication other than by dumping them. For example, a wholesaler informed us that he was now offering medication which was about to expire to customers at discounted prices.

Another set of formal regulations was the Drugs and Cosmetics Act (DCA), 1940, and the Drugs and Cosmetics Rules (DCR), 1945, which regulated the import, manufacture, stocks, distribution, and sales of drugs in India [24]. The primary objective of the DCA was to ensure that the drugs and cosmetics sold in India were safe, effective and conformed to state quality standards. The DCA required that drugs contained in Schedule H (contains all prescription drugs, including antibiotics) and Schedule H1 (contains third and fourth generation antibiotics and tuberculosis drugs) should be sold only on the prescription of a qualified medical practitioner. The Act allows for inspections of a retailer’s documents and sales records against their stocks. However, most retailers did not have a system where they used receipts, often to omit paying taxes. There was lax awareness of and enforcement of Schedule H requirements [25]. In the words of an interviewee, “95% of retailers do not keep the Schedule H documents. Punishment can be given for seven days or one, but this is difficult to implement from the outside” (S24P_Reg Body).

It is also possible to obtain the licence based on at least a couple of years of experience, although this number varies according to the highest educational degree an applicant holds. A key informant explains his experience:

I had to get an experience certificate from the place that I used to work. With that I went to Drug Controller Office in Town X and told them I want to get a license. … Then they ok-ed it and issued me a certificate in a week or two. Then against that certificate, we contacted the C&F agent saying that we want to start this business. After contacting them they sent us medicine. (GP-B_Stockist_1).

In contrast, regulations of some of the H1 drugs were more strictly enforced, including the prohibition of selling psychiatric drugs without prescription and regulations for tuberculosis medication. However, several interviewees were not aware of such restrictions for antibiotics:

Interviewer: Do the people from Drugs Control come to you?

Medical Representative: Yes, they do come.

Interviewer: What do they do?

Medical Representative: They visit. They see if there any expiry medicine in the medical shop. Whether any DOTS prescription has come the shop. This is what they look for. Check whether documents are okay.

Interviewer: Which document?

Medical Representative: License, etc.

Interviewer: How many times a year do they come?

Medical Representative: Some years they visit 2–3 times, some years they don’t visit at all. It depends on their will.

Interviewer: Is there any discussion regarding antibiotics?

Medical Representative: No. And what is the benefit of that discussion?

  (GP-B_MedRep_2)

While antibiotic and other prescription drug sales might be regulated under the DCA and DCR, enforcement and oversight remained a challenge. The lack of any direct laws applicable to IPs further confounded the situation: “There are no laws that apply to RMPs [IPs]. Chemists have regulation regarding DOTS-TB. If any TB patients come to them, he should be instantaneously sent to hospital” (GP-B_MedRep_2).

The DCR regulated licensing, which was another formal regulatory mechanism. Licences in India, according to the nation-wide applicable DCR, must be acquired by all those persons who are engaging in the pharmaceutical business. Licences are numerous and clear information is difficult to filter out. The DCR regulates retail and wholesale licences and includes stockist licences under this banner too. Wholesalers/stockists/distributors can apply for a licence with the Drug Control district office, which is under the auspices of the state government’s Directorate of Drug Control. The person who wants to obtain this licence must have a degree or diploma in pharmacy from a recognised university and a minimum of one-year experience in drug dealing.

In addition to a wholesale licence, stockists (defined here as selling medication from specific companies) required a licence from the Bengal Chemist & Druggist Association (BCDA) in the district to work with companies. According to several interviews, for every company a stockist wanted to work with, the stockist required the BCDA’s approval and the company’s approval (GP-B_MedRep_2). Only seven stockists could be appointed by the BCDA per district, according to an interviewee, with an exception for Kolkata.

A Retail Drug Licence depended on the presence of a registered pharmacist under the State Pharmacist Council, “who must sit an exam every five years to retain their registration” (GP-B_Pharmacy_1). Chemist shops (retailers) needed a separate licence to store and sell veterinary drugs. While a drug store licence required a certified pharmacist to be present for the sale of medication, in practice, several key informants said that a pharmacist is not always present. IPs did not require any type of licence or registration, but the government had started a six-month training programme, which was sometimes understood as a licence, as the following key informant highlighted: “RMP [IP] is given license nowadays. It is happening. There is a RMP [IP] certification now” (GP-B_MedRep_2). Yet, as the interviewee goes on to highlight, the certificate did not actually hold any legal value: “The administration could say: forget the certificate, I will close your shop” (GP-B_MedRep_2).

### 4.4. Informal Regulations

Alongside formal public regulatory mechanisms, actors were seen to regulate one another through several informal mechanisms. This form of informal regulation emerged in different ways: through a tacit support and acceptance for rule breaking, such as through licence sharing amongst small businesses as well as through rulemaking such as through establishing norms for credit systems and for discounts along the chain as explained in an earlier section.

Licence sharing was a mechanism developed in response to formal regulations. As obtaining a licence was sometimes not possible because individuals did not hold any state-recognised training certificate, informal mechanisms were used for business purposes, such as borrowing or sharing a licence from known people:

Stockist: One of my friends has a license. I purchase products using his license number.

Interviewer: Who is that?

Stockist: XY distributors. They have distributor license.

Interviewer: So, you can distribute?

Stockist: I do not distribute. I use the license to purchase. (GP-B_Stockist_2)

This practice was described to us in several interviews, including by medical representatives and stockists as well as an IP who also functioned as a sub-stockist. A similar phenomenon of ‘licence renting’ occurred with pharmacy licences. To open a pharmacy, the proprietor needed proof that the pharmacy would employ a licensed pharmacist. As certified pharmacists were in short supply, their licences could be loaned, for a fee. As one pharmacist explained, in many cases certified personnel were seldom physically present in pharmacies. Instead, their professional credentials were “rented out” for a monthly fee to individuals wishing to open shops without the necessary qualifications—typically local business owners who would pay between 10,000 and 20,000 rupees per month to use the pharmacist’s licence.

These practices were weakly regulated, but some changes were underway. In the past, inspections were often ineffective because officials could be bribed to overlook the absence of licensed pharmacists, and it was common for one pharmacist’s licence to be used across multiple pharmacies for profit. However, respondents noted that government oversight had become stricter, with digital systems now limiting each licence to a single facility. Digitisation was also reported to be increasingly influential in healthcare delivery more broadly, particularly with the growth of telehealth and other digital health applications during the early years of the COVID-19 pandemic (GP-B_Pharmacy).

### 4.5. Power and Legitimacy

Over time, the conception of power in the global value chain literature has broadened from a focus on buyer power to include key suppliers [26]. This leads away from the unipolarity of governance, where power is concentrated in one functional position in the value chain, towards multipolarity, where power might appear in various functional positions. Mapping the antibiotic value chain highlights that power is dispersed along all nodes, albeit not equally. Patients as well as formal physicians create demand by asking for and prescribing specific medication. The value chain is thus influenced by ‘buyer power’, which falls into the category of ‘coercive’ power in which one actor utilises incentives or sanctions directly to compel another actor to act according to their wishes (cf. [27]). Negotiating, offering, or withdrawing discounts, credit systems, and pricing are means of exercising ‘buying power’ at all nodes in the chain. ‘Buyer power’ is resource centred. At the same time, power is transmitted in a more diffuse way. Manufacturers, their distribution companies, and medical representatives promote certain drugs over others thereby influencing demand. Through their role as a source of knowledge, they can also shape perceptions. For example, they can seek to influence notions of quality by promoting branded drugs over generic drugs [26]. Given the lack of formal medical or pharmaceutical training of IPs and many individuals running small-scale drug retail shops, information on quality is often beyond buyers’ scrutiny.

Legitimacy is key to the maintenance of value chains. Both power and legitimacy are continuously (re-)negotiated between actors. However, to Kaplinsky and Morris [1] the legitimacy of power in value chains is expressed through trust. Thus, particularly IPs hold legitimacy as they have gained their patients’ trust, are deeply embedded in their communities or even leaders in their communities. Similarly, the granting of credits with longer and flexible payback and ‘easy’ terms demonstrates high levels of trust among participants, thereby expressing legitimacy. However, it should be noted that legitimacy of power may be undermined by dependencies. Patients, for example, do not have access to formal healthcare, putting IPs in positions of power due to this lack of alternatives. IPs themselves depend on the knowledge transmitted to them through their networks, which demonstrates trust but also a lack of alternative knowledge and information, thereby placing certain actors in positions of greater power in these value chains.

## 5. Discussion

The findings presented above raise important questions for how antibiotic governance and antimicrobial stewardship are conceptualised and operationalised in practice. Rather than operating through clearly bounded actors and enforceable regulations, governance of the antibiotic value chain emerges through overlapping formal and informal arrangements. This discussion explores how these dynamics align with, extend, and complicate existing empirical and policy-oriented accounts, and considers their implications for intervention design.

This complex scenario arises due to several factors. Large unmet healthcare needs and a growing pharmaceutical industry leveraging domestic markets contribute to this dynamic. For instance, Gautham and colleagues [21] highlight the availability, prices, and affordability of antibiotics stocked by informal providers in rural India, emphasising the critical role these providers play in meeting healthcare demands. Similarly, Hutchinson et al. [28] discuss the challenges Ugandan drug shops face in adhering to regulations, illustrating how informal practices are shaped by social and economic contexts. Our findings align with these studies, showing that rule-breaking and informal practices are prevalent due to the necessity of meeting healthcare needs and the flexibility required to operate within these markets.

Our findings indicate that while regulations exist, they are implemented to varying degrees. Depending on the position of the value chain actor, their economic, social, and cultural status, they are subject to varying degrees of formal and informal regulations. Small-scale stockists, retailers, medical representatives, and IPs often fly under the radar of an already overstretched state regulatory and public health system. Several key informants reported that regulations around prescription practices or stocking, distribution, and discarding of antibiotics are not enforced, nor are controls around these issues implemented.

Regulatory infringements, informal and illicit practices, similar to the ones documented in this paper, are also described elsewhere. Researchers have highlighted the widespread rule-breaking related to the stocking of medicines, the prevalence of unlicensed drug shops, and over-the-counter sales of antibiotics in India [11,15,21,29,30]. Our study adds two important aspects to this. The first is a better understanding of the fluidity between formal and informal; the second is recognising that policy attention and target interventions could shift attention away from IPs, who are less powerful and influential, to those who are further up in the value chain. However, further research is needed to substantiate this direction.

Gautham et al. [13], for example, detail several informal practices committed by formal healthcare providers. These include prescribing antibiotics without proper diagnosis, often influenced by patient demands or financial incentives from pharmaceutical companies. Over-the-counter sales of prescription medications by licensed retailers further bypass regulatory requirements. Their study further found that formal providers may also collaborate with informal providers, sharing prescriptions and medical advice, which may lead to inappropriate medication use. Our study complements this finding from the perspective of IPs, highlighting the benefits of such collaboration. Formal healthcare providers are an important source of information and medical knowledge for IPs. Contact with primary healthcare providers has helped IPs build better referral structures where the IP feels more comfortable referring patients or approaching a physician for help.

Our study revealed the fluidity between formal and informal and the challenge of neat compartmentalisation. Antimicrobial stewardship guidelines and intervention often target specific groups. However, some of these groups are less homogeneous than suggested in national-level policymaking. One example of this is the role of retail shops in the value chain. Brhlikova and colleagues [30] estimate that 500,000 to 600,000 pharmacists practiced in India between 2000 and 2005. In rural areas, small retail shops dotted the landscape, many of which were part of the state-wide associations including the Bengal Chemist & Druggist Association (BCDA) and Progressive Chemist & Druggist Association (PCDA). Not all retail shops hold licences, and they are small, but their power lies in numbers and their ability to organise. Some are run by IPs, which means these individuals double as chemist or pharmacist. The availability of drug retail shops is essential for urban and rural areas, yet their function and clientele can be drastically different. Kotwani’s research further supports our observations regarding the openness and flexibility of licence requirements [16]. Wholesalers and other value chain actors often navigate a regulatory landscape that is not as rigid as it appears, allowing for a range of practices that may not strictly adhere to formal regulations.

Market mechanisms that define the value chain were also on a spectrum of formal–informal. Discounts and credit systems are regularly negotiated between those selling and those buying antibiotics. Some retailers have more formalised credit systems with end-of-year accounting while others work on a case-to-case system. Formal regulatory authorities often adhere to unwritten, yet common-sensical rules due to practical constraints. Gautham and colleagues found that drug inspectors, aware of illegal antibiotic sales, are reluctant to take action without higher orders, as higher authorities are concerned about restricting access to healthcare and the political consequences of prosecuting IPs.

The term ‘informal’ presents a political challenge in recognising the role of IPs within the value chain. Acknowledging their contribution is crucial as it reflects the realities of healthcare delivery for humans and animals in rural and peri-urban settings in West Bengal and beyond. Neglecting their role negates the significant part they play in these communities. The analysis of the value chain presented here supports an indicative need to incorporate IPs into formal health strategies to enhance healthcare outcomes and ensure more comprehensive stewardship practices. Guidelines for antibiotic use present a cost-effective alternative to provide easily accessible information, which is less prone to influence from specific producers and their distribution mechanisms. One important finding is that guidelines become scarcer the further one moves towards the end-consumer in the value chain. The lack of formal regulation allows for flexibility in the type of roles IPs take on and the networks they create, including market-based mechanisms such as credit systems. However, the absence of regularly updated guidelines means that IPs rely on information provided by medical representatives or prescriptions from primary care physicians to learn of new medications and what they may be used for.

What these findings highlight is that less power may lie with IPs than is often attributed to them in the policy discourse. While those at the bottom rungs of the value chain, such as medical representatives and IPs, are often held responsible for antibiotic misuse, our study highlights that actors higher up in the chain—wholesalers with their own representatives, manufacturers, PD companies, and marketing companies—are not as well-known and often escape scrutiny. This oversight allows significant contributors to antibiotic distribution and misuse to operate without adequate regulation. A preliminary implication of our findings is that there is a need to identify and engage with key power nodes within the value chain, such as the association of chemists, to implement more effective antimicrobial stewardship strategies. Additionally, understanding the pricing mechanisms and market dynamics is crucial to rationalising antibiotic use and ensuring that interventions target the appropriate actors within the value chain. By addressing these higher-level nodes, we can create a more comprehensive and effective approach to managing antibiotic distribution and use.

## 6. Study Limitations

Like all research, our study has several limitations. While the study focused on two districts in West Bengal with a relatively small sample of key informant interviews, this was intentional given the exploratory and qualitative design, which prioritises depth of insight over statistical generalisability. We therefore refrain from making claims beyond the specific context studied and instead offer findings that illuminate processes likely relevant to similar settings in India and beyond.

Participant selection was based on purposeful and snowball sampling to capture a broad range of actors across the pharmaceutical value chain, but we recognise that this approach may introduce selection bias, as those who agreed to be interviewed may differ systematically from those who declined. Qualitative research data can be influenced by hidden agendas, assumptions, and the external perspective of the research team. As researchers focusing on antibiotic use and stewardship, we inherently support antibiotic reform as beneficial and in the public interest. Participants aware of their potentially inappropriate antibiotic practices may have introduced social desirability bias. Additionally, our findings are shaped by interactions between the research team, translators, and interviewees, meaning alternative interpretations may exist.

Furthermore, translation between languages can shape meaning and nuance. We attempted to address these challenges by working closely with bilingual or polyglot local partners, reviewing translated transcripts carefully, and discussing key terms and interpretations collaboratively within the team. Members of the research team, who were fully aware of the research project and who also helped in developing the interview guides, were there to help with translation where needed. To mitigate biases, we used non-leading open-ended questions, collected data from diverse sources, and collaborated with local partners familiar with the research context to provide a more internal perspective, thereby attempting to triangulate our findings.

## 7. Conclusions

In this study, we examined the key components that shape governance of drugs and antibiotic value chains within a domestic political context, drawing on the framework outlined by Kaplinsky and Morris. Our analysis reveals critical gaps in understanding of the value chain that are crucial for designing effective antimicrobial stewardship strategies. As demonstrated in earlier sections, the coordination and regulation of the antibiotic value chain is not controlled by a single entity. Instead, multiple and densely populated nodal points contribute to its governance, encompassing both formal structures and informal mechanisms. Actors within the value chain do not conform to neat categories of chemists, independent pharmacists, wholesalers or stockists, often holding multiple roles simultaneously. Furthermore, while learning mechanisms exist, there is a significant disparity between those with access to up-to-date information and those without. This is a highly competitive and autonomous market governed by formal as well as its own informal regulations.

Mapping the antibiotic value chain reveals a complicated interplay of diverse actors, their vested interests, and patient needs, which is defined by an asymmetry of information between the possessors of specialised knowledge and expertise, much needed local-contextualised knowledge held by informal providers, and patients who lack access to the formal healthcare system. What this analysis highlights are the inadequacies of the binary formal–informal, attempts to neatly categorise actors, and the need to engage closely with local contexts and aspects that are deemed ‘informal’ in order to design holistic antimicrobial stewardship that actually reaches all concerned parties because informalities extend far beyond a group of providers and encompass the entire value chain of antibiotics.

## Figures and Tables

**Table 1:** Governance dimensions of antibiotic value chains according to Kaplansky and Morris, 2002 [1].

Actors and actor relationships	Mapping of relevant actors (e.g., manufacturers, wholesalers, prescribers, pharmacies, informal providers, regulators) and how they relate to one another.
Nature of relationships	Examination of why actors establish specific relationships (e.g., market access, rent opportunities, resource-sharing) and how these influence roles, responsibilities, and flows of antibiotics within the chain.
Formal regulation	The legislative and policy frameworks (e.g., drug licensing, prescription requirements, stewardship guidelines) that define the formal boundaries and obligations of antibiotic production, distribution, and use.
Informal mechanisms	Informal practices that supplement or bypass formal regulation, such as the role of unlicensed providers, informal retail channels, or social norms shaping antibiotic access and use.
Power and legitimacy	The ways actors make, break, and negotiate rules, and the mechanisms by which rules are enforced, contested, or legitimised, reflecting underlying power relations in the value chain.

**Table 2:** Formal regulations governing antibiotic supply chains in peri-urban India and their circumvention in practice.

Formal Regulation	Key Provisions/What It Tries to Do	Supply Chain Tiers It Formally Targets	How Actors Break, Bend, or Bypass It
**Goods & Services Tax (GST) Act, 2017**	Only GST-registered businesses may buy medicines from companies/wholesalers. Requires separate accounting for GST vs. non-GST customers. Manufacturers and C&F agents may not accept expired stock (to stop illicit re-sale).	Manufacturers → C&F agents → stockists/wholesalers	Retailers still sell to IPs via “unregistered sales”. With no authorised route to return leftovers, near-expiry antibiotics are off-loaded at big discounts or simply dumped.
**Drugs & Cosmetics Act (1940) + Rules (1945)—esp. Schedules H and H1**	Import, manufacture, stocking, distribution, and sale of drugs. Schedule H/H1 antibiotics and TB/psychotropic drugs must be sold only on a qualified practitioner’s prescription. Inspectors may check sales records vs. physical stock.	Retail pharmacies, wholesalers, prescribers	Most retailers do not keep Schedule-H registers; prescriptions rarely demanded. Inspectors visit irregularly and focus on TB DOTS forms or expiry dates, not antibiotic sales. IPs continue to purchase and dispense antibiotics.
**Licensing under the Drugs & Cosmetics Rules (retail, wholesale, stockist)**	Wholesale licence: pharmacy degree/diploma + ≥1 yr experience; issued by state Drug Control. Each stockist also needs Bengal Chemist & Druggist Association (BCDA) approval Retail drug licence requires a registered pharmacist to be on the premises at all times	Stockists, wholesalers, retailers/chemists	Licence sharing: stockists buy under a friend’s distributor number; pharmacists lease their registration to shops for a fee. Pharmacists are not always present or move around shops for the purpose of inspection. IPs act as sub-stockists or dual chemist-prescribers without any licence.
**BCDA quota on stockists (≤7 per district)**	Aims to limit the number of authorised stockists a company may appoint in one district, ostensibly to curb oversupply and ease supervision.	Stockists	Companies and distributors sidestep the cap by using sub-stockists (often IPs or medical reps) who hold no BCDA approval but operate as micro-wholesalers closer to villages.

## Data Availability

The dataset analysed during the current study is available from the corresponding author on reasonable request.

## References

[1] Kaplinsky R., Morris M. (2002). A Handbook for Value Chain Research.

[2] IHME (2023). The Burden of Antimicrobial Resistance (AMR) in India. IHME. https://www.healthdata.org/sites/default/files/2023-09/India.pdf.

[3] GoI, WHO National Action Plan on Antimicrobial Resistance (NAP-AMR) 2017–2021. Ministry of Health and Family Welfare, Government of India and World Health Organization Country Office for India. April 2017. https://www.who.int/publications/m/item/india-national-action-plan-on-antimicrobial-resistance-(nap-amr)-2017-2021.

[4] Klein E.Y., Milkowska-Shibata M., Tseng K.K., Sharland M., Gandra S., Pulcini C., Laxminarayan R. (2021). Assessment of WHO antibiotic consumption and access targets in 76 countries, 2000–2015: An analysis of pharmaceutical sales data. Lancet Infect. Dis..

[5] Browne A.J., Chipeta M.G., Haines-Woodhouse G., Kumaran E.P.A., Hamadani B.H.K., Zaraa S., Henry N.J., Deshpande A., Reiner R.C., Day N.P.J. (2021). Global antibiotic consumption and usage in humans, 2000–2018: A spatial modelling study. Lancet Planet. Health.

[6] Das J., Daniels B., Ashok M., Shim E.Y., Muralidharan K. (2022). Two Indias: The structure of primary health care markets in rural Indian villages with implications for policy. Soc. Sci. Med..

[7] Gautham M., Shyamprasad K.M., Singh R., Zachariah A., Singh R., Bloom G. (2014). Informal rural healthcare providers in North and South India. Health Policy Plan.

[8] Government of India (2016). Health in India.

[9] Morgan D.J., Okeke I.N., Laxminarayan R., Perencevich E.N., Weisenberg S. (2011). Non-prescription antimicrobial use worldwide: A systematic review. Lancet Infect. Dis..

[10] Hennessey M., Ebata A., Samanta I., Mateus A., Arnold J.C., Day D., Gautham M., Alarcon P. (2023). Pharma-cartography: Navigating the complexities of antibiotic supply to rural livestock in West Bengal, India, through value chain and power dynamic analysis. PLoS ONE.

[11] Bloom G., Henson S., Peters D.H. (2014). Innovation in regulation of rapidly changing health markets. Glob. Health.

[12] Auta A., Hadi M.A., Oga E., Adewuyi E.O., Abdu-Aguye S.N., Adeloye D., Strickland-Hodge B., Morgan D.J. (2019). Global access to antibiotics without prescription in community pharmacies: A systematic review and meta-analysis. J. Infect..

[13] Ebata A., Gautham M., Jung A.S., Hennessey M., Bhattacharyya S., Bloom G. (2025). Understanding the complex knowledge economy toward antimicrobial stewardship in West Bengal, India. SSM-Health Syst..

[14] Gautham M., Spicer N., Chatterjee S., Goodman C. (2021). What are the challenges for antibiotic stewardship at the community level? An analysis of the drivers of antibiotic provision by informal healthcare providers in rural India. Soc. Sci. Med..

[15] Nair M., Tripathi S., Mazumdar S., Mahajan R., Harshana A., Pereira A., Jimenez C., Halder D., Burza S. (2019). “Without antibiotics, I cannot treat”: A qualitative study of antibiotic use in Paschim Bardhaman district of West Bengal, India. PLoS ONE.

[16] Kotwani A., Bhanot A., Singal G.L., Gandra S. (2022). Marketing and Distribution System Foster Misuse of Antibiotics in the Community: Insights from Drugs Wholesalers in India. Antibiotics.

[17] Mentzer J.T., DeWitt W., Keebler J.S., Min S., Nix N.W., Smith C.D., Zacharia Z.G. (2001). Defining Supply Chain Management. J. Bus. Logist..

[18] Gereffi G., Humphrey J., Sturgeon T. (2005). The Governance of Global Value Chains. Rev. Int. Political Econ..

[19] Kiambi S., Onono J.O., Kang’ethe E., Aboge G.O., Murungi M.K., Muinde P., Akoko J., Momanyi K., Rushton J., Fèvre E.M. (2020). Investigation of the governance structure of the Nairobi dairy value chain and its influence on food safety. Prev. Vet. Med..

[20] Government of India Top 10 Most Populated States in India with Highest Population 2025. https://www.census2011.co.in/facts/topstatepopulation.html.

[21] IIPS (2019). District Level Household and Facility Survey (DLHS-4), 2012–2013 (Updated 2019).

[22] Gautham M., Miller R., Rego S., Goodman C. (2022). Availability, Prices and Affordability of Antibiotics Stocked by Informal Providers in Rural India: A Cross-Sectional Survey. Antibiotics.

[23] McCubbin K.D., Anholt R.M., de Jong E., Ida J.A., Nóbrega D.B., Kastelic J.P., Conly J.M., Götte M., McAllister T.A., Orsel K. (2021). Knowledge Gaps in the Understanding of Antimicrobial Resistance in Canada. Front. Public Health.

[24] Centre for Market Research & Social Development (2023). Survey of Pharma Clusters.

[25] Ministry of Health & Family Welfare, Government of India The Drugs and Cosmetics Act, 1940. The Drugs and Cosmetics Rules, 1945 (Amended up to 31 December 2016) December 2016. https://cdsco.gov.in/opencms/export/sites/CDSCO_WEB/Pdf-documents/acts_rules/2016DrugsandCosmeticsAct1940Rules1945.pdf.

[26] Mathew P., Thomas S.A., Chandy S.J. (2022). The role of Schedule H1 and Red Line campaign in improving antibiotic use in India. J. Fam. Med. Prim. Care.

[27] Dallas M.P., Ponte S., Sturgeon T.J. (2019). Power in global value chains. Rev. Int. Political Econ..

[28] Dahl R.A. (1957). The concept of power. Behav. Sci..

[29] Hutchinson E., Hansen K.S., Sanyu J., Amonya L.P., Mundua S., Balabanova D., Clarke S., Kitutu F.E. (2023). Is it possible for drug shops to abide by the formal rules? The structural determinants of community medicine sales in Uganda. BMJ Glob. Health.

[30] Brhlikova P., Harper I., Jeffery R., Rawal N., Subedi M., Santhosh M. (2011). Trust and the regulation of pharmaceuticals: South Asia in a globalised world. Glob. Health.

